# Interstitial Telomeric Sequences (ITS) and major rDNA mapping reveal insights into the karyotypical evolution of Neotropical leaf frogs species (*Phyllomedusa,* Hylidae, Anura)

**DOI:** 10.1186/1755-8166-7-22

**Published:** 2014-03-06

**Authors:** Daniel Pacheco Bruschi, Miryan Rivera, Albertina Pimentel Lima, Ailín Blasco Zúñiga, Shirlei Maria Recco-Pimentel

**Affiliations:** 1 Departamento de Biologia Estrutural e Funcional, Instituto de Biologia, Universidade Estadual de Campinas (UNICAMP), 13083-863 Campinas, São Paulo, Brazil; 2 Escuela de Ciencias Biológicas, Pontifícia Universidad Católica Del Ecuador, Quito, Ecuador; 3 Instituto Nacional de Pesquisas da Amazônia (INPA), 69060-001 Manaus, AM, Brazil

**Keywords:** *Phyllomedusa*, Karyotypes, rDNA, Interstitial telomeric sequences (ITS)

## Abstract

**Background:**

The combination of classical cytogenetics with molecular techniques represents a powerful approach for the comparative analysis of the genome, providing data for the systematic identification of chromosomal homologies among species and insights into patterns of chromosomal evolution within phylogenetically related groups. Here, we present cytogenetic data on four species of Neotropical treefrogs of the genus *Phyllomedusa* (*P. vaillantii*, *P. tarsius*, *P. distincta*, and *P. bahiana*), collected in Brazil and Ecuador, with the aim of contributing to the understanding of the chromosomal diversification of this genus.

**Results:**

With the exception of *P. tarsius*, which presented three telocentric pairs, all the species analyzed had conservative karyotypic features. Heterochromatic patterns in the genomes of these species revealed by C-banding and fluorochrome staining indicated the presence of a large number of non-centromeric blocks. Using the Ag-NOR method and FISH with an rDNA 28S probe, we detected NOR in the pericentromeric region of the short arm of pair 7 in *P. vaillantii*, pair 1 in *P. tarsius*, chromosomes 1 and 9 in *P. distincta*, and in chromosome 9 in *P. bahiana*, in addition to the presence of NOR in one homologue of chromosome pair 10 in some individuals of this species. As expected, the telomeric probe detected the terminal regions of the chromosomes of these four species, although it also detected Interstitial Telomeric Sequences (ITS) in some chromosomes of the *P. vaillantii*, *P. distincta* and *P. bahiana* karyotypes.

**Conclusion:**

A number of conservative chromosomal structures permitted the recognition of karyotypic homologies. The data indicate that the presence of a NOR-bearing chromosome in pair 9 is the plesiomorphic condition in the *P. burmeisteri* group. The interspecific and intraspecific variation in the number and location of rDNA sites reflects the rapid rate of evolution of this character in *Phyllomedusa*. The ITS detected in this study does not appear to be a remnant of structural chromosome rearrangements. Telomeric repeats were frequently found in association with heterochromatin regions, primarily in the centromeres, which suggests that (TTAGGG)n repeats might be an important component of this heterochromatin. We propose that the ITSs originated independently during the chromosomal evolution of these species and may provide important insights into the role of these repeats in vertebrate karyotype diversification.

## Background

Comparative cytogenetic studies provide scenarios of the chromosomal evolution of related taxa and represent an important approach to the identification of chromosomal homologies among species [1]. In many organisms, karyological features have been widely accessed by classical methods, and advances in molecular cytogenetics based on Fluorescence *in situ* hybridization (FISH) experiments have resulted in improved chromosomal mapping of large numbers of sequences and permitted the study of chromosomal variation.

The ribosomal RNA gene, which is a repetitive DNA sequence that is organized in tandem, is widely used in chromosomal investigations and provides a good chromosomal marker for comparative cytogenetic studies [2]. This sequence shows several features of ‘hotspots’ of chromosomal recombination because it consists of a clustered organization of repeats and is frequently located in pericentromeric and subtelomeric regions [3]. A high rate of mutation/homogenization of intergenic spacer regions is observed (e.g. see references [4-7]), and these modifications have an important role in chromosomal reorganization during karyotype evolution. The association of the NOR repositioning events due to the presence of transposable elements has already been noted [8,9].

Chromosomal mapping of telomeric sequences has been widely used to identify chromosomal rearrangements among karyotypes of vertebrates and to detect fusion and/or fission, inversion or translocation events [10-16]. Many recent studies have emphasized the important role of this sequence in chromosomal evolution [17-19], and many studies have reported that sequences related to telomeric sequences form a component of satellite DNA [20-22].

Cytogenetic studies have demonstrated the presence of interstitial telomeric sequences (ITS) in many phylogenetic groups (for references, see reference [23]). In most cases, these sequences are associated with heterochromatin regions that do not appear to represent remnants of ancient chromosomal rearrangements [24-29]. Non-telomeric repeats of the sequence (TTAGGG)n in heterochromatin regions or in the margins of these blocks, which have been termed ‘het-ITS’ are easily detected in FISH experiments [23]. However, fine-scale studies in mammals have also documented the extensive occurrence of short telomeric repeats within the internal regions of chromosomes (s-ITSs) [18,30-32], and, according to Ruiz-Herrera [19], this feature is presumably present in all vertebrate species. The presence of s-ITS in other vertebrates could be underestimated due to the presence of fewer repeats; such repeats may not be detectable at the resolution of conventional FISH experiments.

The *Phyllomedusa* genus is an interesting group within which to conduct comparative cytogenetic analyses. In addition to the fact that the intrageneric relationships of some of the species remain unclear, this Neotropical treefrog genus raises many taxonomic questions at the species level [33]. The genus currently includes 30 species [34]. Molecular phylogenetic inferences support the presence of four species groups [33]: the *P. hypochondrialis* group [35], the *P. tarsius* group [36], the *P. burmeisteri* group [37] and the *P. perinesos* group [38]. The species *P. atelopoides*, *P. bicolor*, *P. boliviana*, *P. vaillantii*, *P. sauvaggi*, and *P. tomopterna*[33,34] are not included in any of these groups. Cytogenetic data show extensive multiple NOR [39-44] and interspecific NOR variation [40,41,43,45] in the species that have been karyotyped.

Our goal in this work was to investigate the karyotypes of four species of the *Phyllomedusa* genus: *P. vaillantii*, species that remain unassigned to any species group, *P. tarsius,* include in the *P. tarsius* group and *P. distincta* and *P. bahiana* which are included in the *P. burmeisteri* group. We used multiple chromosomal markers to better understand the chromosomal evolution of this genus. In addition to providing insights concerning the evolution of rDNA clusters, we report an interesting distribution pattern of non-terminal telomeric repeats in karyotypes of this genus.

## Results

**Figure 1:**
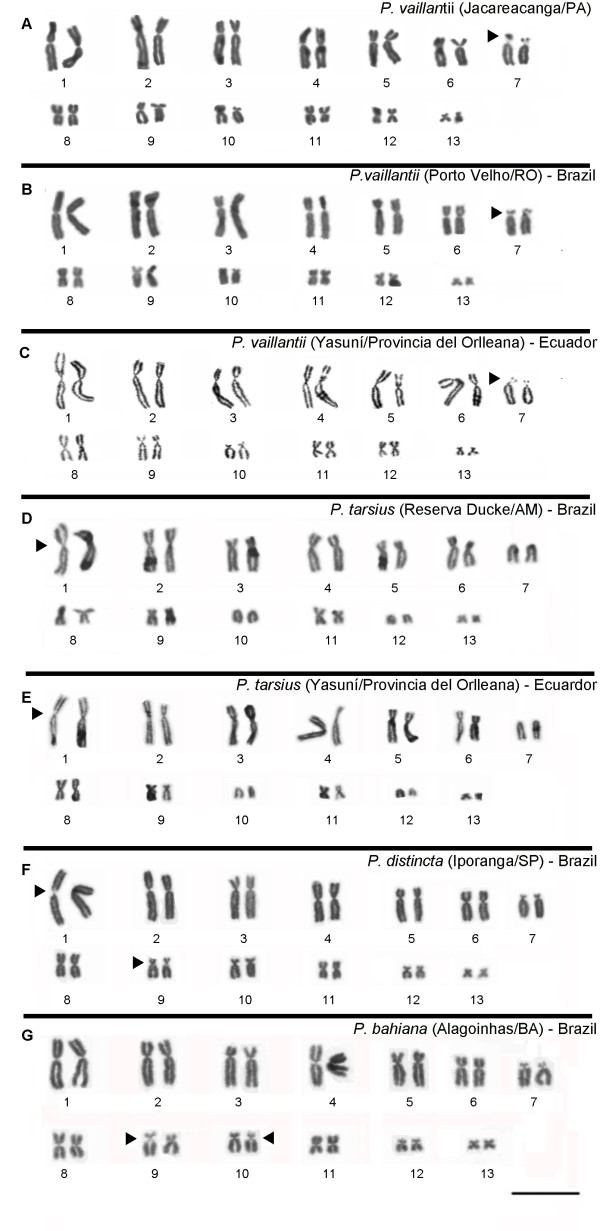
**Giemsa-stained karyotypes of *P. vaillantii *from (A) Jacareacanga/PA, Brazil, (B) Porto Velho/RO, Brazil and (C) Yasuní/ Provincia del Orlleana, Ecuador; *P. tarsius *from (D) Reserva Ducke/AM, Brazil and from (E) Yasuní/Provincia del Orlleana, Ecuador; (F) *P. distincta *from Iporanga/SP, Brazil and (G) *P. bahiana *from Alagoinhas/BA, Brazil.** The arrowhead indicates secondary constrictions. Bar = 3 μm.

**Figure 2:**
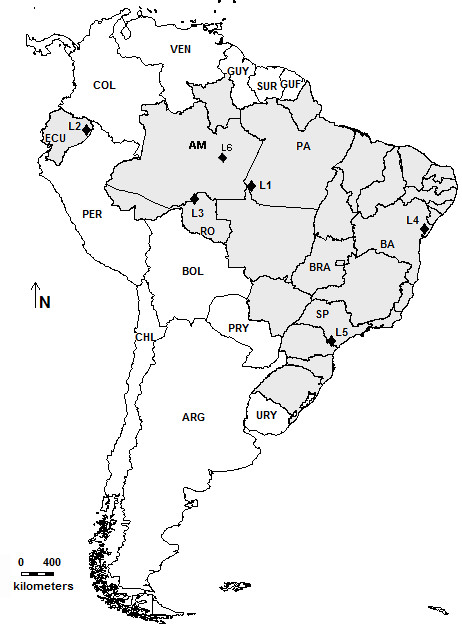
**Topographic map of South America showing sampling localities throughout Brazil and Ecuador for populations included in this study.** L1: Jacareacanga/PA, Brazil; L2: Yasuní/Provincia del Orlleana, Ecuador; L3: Porto Velho/RO, Brazil; L4: Alagoinhas/BA, Brazil; L5: Iporanga/SP, Brazil; L6: Reserva Ducke/AM, Brazil.

The chromosome diploid number in all species karyotyped showed 26 chromosomes. With the exception of *P. tarsius*, which showed three telocentric pairs (pairs 7, 10 and 12), the remaining karyotypes consisted of four metacentric pairs (1, 4, 8 and 11), six submetacentric pairs (2, 3, 5, 6, 12 and 13) and three subtelocentric pairs (7, 9 and 10) (Figure 1A-G). Secondary constrictions was observed in the pericentromeric region of the short arm of pair 7 in *P. vaillantii* karyotype, in the pair 1 in *P. tarsius*, pairs 1 and 9 in *P. distincta*, and in chromosomes 9 in *P. bahiana* karyotype, besides secondary constriction in one homologue of the pair 10 (see Figure 2).

**Figure 3:**
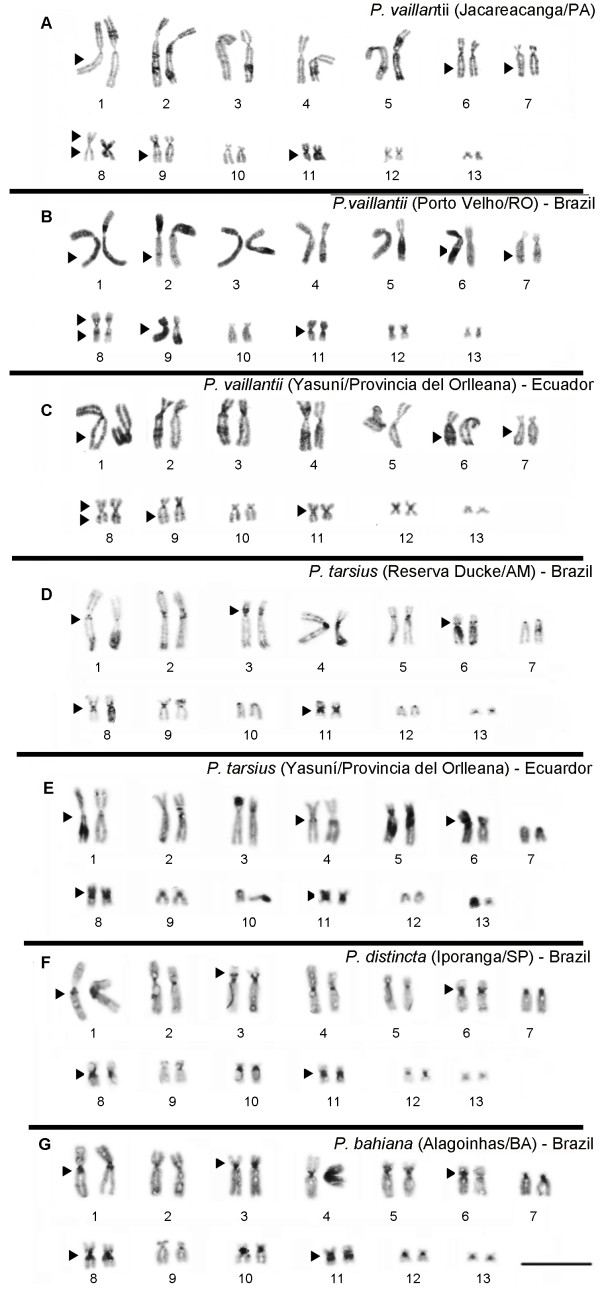
**Karyotypes defined by C-banding of *P. vaillantii *from (A) Jacareacanga/PA, Brazil, (B) Porto Velho/RO, Brazil and (C) Yasuní/ Provincia del Orlleana, Ecuador; *P. tarsius *from (D) Reserva Ducke/AM, Brazil and from (E) Yasuní/Provincia del Orlleana, Ecuador; (F) *P. distincta *from Iporanga/SP, Brazil and (G) *P. bahiana *from Alagoinhas/BA, Brazil.** The arrowhead indicates interstitial heterochromatin blocks. Bar = 3 μm.

C-banding detected centromeric heterochromatin in all chromosome pairs, with difference in amount of heterochromatin among pairs: some chromosomes pairs exhibits weak and almost absence heterochromatin block while others pairs exhibits salient marker in this region. Non-centromeric heterochromatin blocks were widely found in *P. vaillantii* chromosomes of the three populations sampled (Figure 3A-C). Remarkable interstitial heterochromatin blocks were detected in both arms of the chromosomes of pair 8 in addition to interstitial C-bands in homologs of pairs 1, 6, 7, 9 and 11 (Figure 3A-C). The karyotype of *P. tarsius* showed C-bands in the pericentromeric region of the short arm of chromosome pairs 3 and 6 and in the long arms of pairs 1, 4 and 11 (Figure 3D-E). The same C-banding pattern was observed in Brazilian and in Ecuadorian populations. Heterochromatin was also detected in both arms of the chromosomes of pair 8 and in the pericentromeric region (Figure 3D-E). *P. distincta* exhibited the presence of C-bands in the pericentromeric region of the short arms of chromosome pairs 3 and 6 and in the long arms of pairs 1 and 11 (Figure 3F). In this species, a C-positive pericentromeric block was also detected in both arms of the chromosomes of pair 8 (Figure 3F). The heterochromatin pattern of *P. bahiana* revealed C-bands in the pericentromeric region of the short arms in homologs of pairs 3 and 6 and in the long arms of pairs 1 and 11 (Figure 3G). The homologs of pair 8 exhibited pericentromeric blocks in both arms.

**Figure 4:**
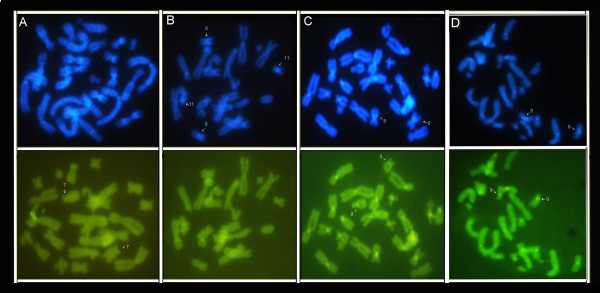
**DAPI staining (top) and Mitramycin (bottom) after C-banding in chomosomes of the (A) *P. vaillantii*, (B) *P. tarsius*, (C) *P. distincta *and (D) *P. bahiana*.** The arrows highlighters specific chromosome pairs of each species according to results section.

The use of base-specific fluorochrome staining after C-banding of chromosomes improved the detection of heterochromatin patterns and revealed additional interesting features of the karyotypes studied. In the case of *P. vaillantii*, pericentromeric C-bands showed DAPI-positive patterns (pairs 1, 6, 7, 8 and 11) (Figure 4A, top). In addition to the blocks detected by the C-banding method, bright interstitial signals were observed. Mithramycin staining resulted in brilliant signals in regions coincident with secondary constrictions in the short arms of the homologs of pair 7 visualized by Giemsa staining (Figure 4A, bottom). In metaphase chromosomes of *P. tarsius*, DAPI staining exhibited a pattern coincident with C-banding, with outstanding bright signals in the heterochromatin of both arms of chromosome pairs 8 and 11 (Figure 4B), whereas MM staining produced only a modest signal coincident with the region containing secondary constrictions in the homologs of chromosome pair 1 (Figure 4B, bottom). The DAPI pattern showed centromeric fluorescence in almost all chromosomes of *P. distincta* in addition to some fluorescence in the region of the secondary constriction in chromosome pair 9 (Figure 4C). In chromosome pair 8, a brilliant signal was evident in the pericentromeric block of the long arm (Figure 4C). The MM pattern was evident in the centromeres of almost all chromosomes as well as in the region coincident with the secondary constriction in the short arms of the homologs of pair 9 (Figure 4C, bottom). The heterochromatin of the centromeres of chromosome pairs 5 and 13 did not show a strong signal with any fluorochrome staining (Figure 4C). Specimens of *P. bahiana* exhibited relatively weak fluorescence in the centromeric regions of the majority of the chromosome pairs (Figure 4D); in these specimens, the pericentromeric heterochromatin was more easily detected by C-banding. The secondary constrictions in the short arm of chromosome pair 9 showed fluorescence signals by MM-staining, and some centromeres were stained (Figure 4D).

**Figure 5:**
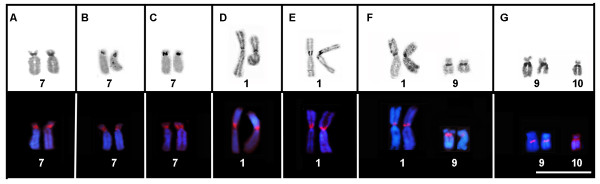
**NOR-bearing chromosome pairs submitted to silver impregnation using Ag-NOR method (top) and hybridized with 28S rDNA probe (bottom): *P. vaillantii *from (A) Jacareacanga/PA, Brazil, (B) Porto Velho/RO, Brazil and (C) Yasuní/ Província de Orllana, Ecuador; *P. tarsius *from (D) Reserva Ducke/AM, Brazil and from (E) Yasuní/Província de Orllana; (F) *P. distincta *and (G) *P. bahiana*.** Bar = 3 μm.

In all karyotypes, the secondary constrictions observed in conventional Giemsa staining were coincident with NOR sites detected by the Ag-NOR method and this was confirmed by FISH experiments (Figure 5). In the three sampled populations of *P. vaillantii*, NORs were detected in the pericentromeric region of the short arm of chromosome pair 7 (Figure 5A-C). The Ag-NOR method also revealed NOR in the pericentromeric region of the short arm of chromosome pair 1 in *P. tarsius* from Reserva Ducke (Manaus, Brazil) and from Yasuní (Província del Orellana, Ecuador) (Figure 5D-E). Two NOR-bearing chromosome pairs were detected in all specimens of *P. distincta* analyzed; the NORs were located in the pericentromeric region of the short arm of chromosome pair 1 and in the pericentromeric region of the long arm of chromosome pair 9 (Figure 5F). Specimens of *P. bahiana* displayed a NOR that was fixed in the pericentromeric region of the long arms of the chromosomes of pair 9 (Figure 5G). In this population, two specimens presented one additional NOR in one of the homologs of chromosome pair 10 (Figure 5G). This conditional was also detected in FISH experiments using an rDNA 28S probe.

**Figure 6:**
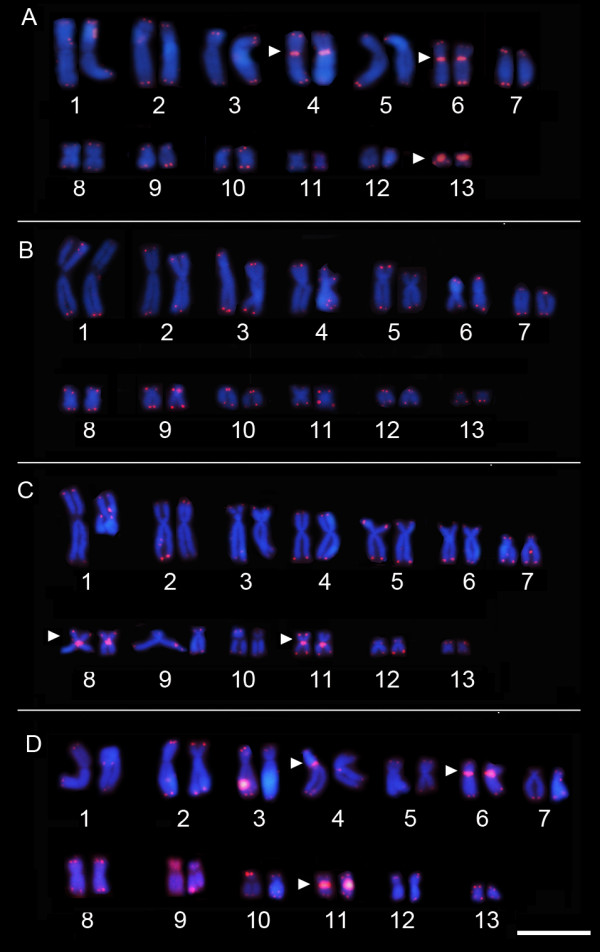
***In situ *hybridization with the telomeric probe in karyotypes of (A*) P. vaillantii*, (B) *P. tarsius, *(C) *P. distincta *and (D) *P. bahiana*.** The arrowhead indicates interstitial telomeric sequence (ITS) adjacent to constitutive heterochromatin (het-ITSs). Bar = 3 μm.

The telomeric probe hybridized to all telomeres in the chromosomes of all karyotypes analyzed but showed a differential pattern of interstitial signals in the four species examined. The *P. vaillantii* karyotype exhibited conspicuous signs of ITS in the centromeric regions of chromosome pairs 4 and 6 (Figure 6A), whereas a brighter hybridization signal was detected in the short arms of the homologs of chromosome pair 13 (Figure 6A), the complete short arm being marked by the telomeric probe. Although *P. tarsius* did not exhibit interstitial signals in any chromosomes, the telocentric morphology of chromosome pairs 7, 9 and 10 was most evident through this approach (Figure 6B). ITS was also detected in the centromeric regions of chromosome pairs 8 and 11 of *P. distincta* (Figure 6C). Finally, in the karyotype of *P. bahiana*, the telomeric probe hybridized to the centromeric region of chromosomes pairs 4 and 6, and stronger hybridization that extended to the pericentromeric region of the arms was detected in the centromeric region of chromosome pair 11 (Figure 6D).

## Discussion

Chromosomal analysis of four representatives of the genus *Phyllomedusa* revealed conservative karyotypic features, including primarily diploid chromosome number (2n = 26), a finding that is consistent with previous reports [39-46]. One special case that showed a deviation in chromosome number was *P. tetraploidea* (2n = 52) [47], a polyploid species with a karyotype clearly derived from 2n = 26.

The four species of the *Phyllomedusa* genus karyotyped in this study showed conserved chromosomal morphology. Despite the different numeric classification of some chromosome pairs among karyotype descriptions in the literature, it is possible to recognize homologies among almost all chromosomes. In this context, the pair 7 subtelocentric and 8 metacentric describes here correspond to, respectively, the pairs 8 subtelocentric and 7 metacentric of the karyotype described by Gruber et al. [44] and Barth et al. [42]. The chromosomal classification used in this study is based on the karyotype ordination described by Bruschi et al. [43] and Bruschi et al. [45]. However, this difference in chromosomal ordination did not represent a real cytogenetic variation among karyotypes of the *P. distincta* and *P. bahiana*.

The telocentric chromosome pairs observed in the *P. tarsius* karyotype are apparently restricted to species of the *P. tarsius* group. These karyotypic traits were detected in *P. camba,* another species within this phenetic group, and this condition was noted as indicative of a possible karyological synapomorphy for the *P. tarsius* group [41]. Currently, the *P. tarsius* group includes *P. camba*, *P. neildi*, *P. trinitatis* and *P. tarsius*[34], although cytogenetic data from *P. neildi* and *P. trinitatis* are necessary to confirm this hypothesis. However, the chromosome complement of one population of *P. tarsius* from Peru described by Bogart [47] showed exclusively bi-armed pairs. If the population of *P. tarsius* from Peru analyzed in that study corresponds to the same taxonomic unit as the Brazilian and Ecuadorian populations analyzed here, the synapomorphy proposed by Paiva et al. [41] should be reevaluated. We have not discarded the possibility that this inconsistency may be due to misidentification of the Peruvian population.

The study of the distribution of heterochromatin in the genomes of these four species using the C-banding method and fluorochrome-specific base staining revealed the presence of a considerable number of non-centromeric blocks, primarily in the *P. vaillantii* karyotype. The C-banding pattern of *P. distincta* observed here is coincident with the arrangement of heterochromatic regions described by Gruber et al. [44]. The metacentric chromosome pair 8 showed pericentromeric heterochromatin in both arms in karyotypes of the *P. tarsius*, *P. distincta* and *P. bahiana* analyzed here and could be a chromosome marker in karyotype of this species. In the *P. vaillantii*, the pair 8 showed C-positive band in interstitial region, noticeable feature of this karyotype.

Fluorochrome staining after C-banding permitted the identification of an AT-rich class of heterochromatin in the C-positive blocks of the karyotype of *P. vaillantii* as well as in the centromeric regions of the majority of *P. tarsius* and *P. bahiana* chromosomes. Consistent with previous suggestions, the MM markers were coincident with secondary regions. Similar brilliant signals with DAPI and MM staining in the centromeric regions of the majority of chromosome pairs in the *P. distincta* karyotype and in some chromosome pairs of *P. bahiana* could be explained by the presence of similar amounts of AT and GC bases within the repetitive sequences present in this region of heterochromatin. Similar labeling was reported in *Eleutherodactylus atkinsi*[14], *E. pantone* and *Pristimantis terraebolivaris* and in *Sphaenorhynchus lacteus*[16].

*P. tarsius* showed rDNA cluster detected in chromosome 1, as well as, *P. camba,* another species in the *P. tarsius* group, also showed NOR in same chromosome pair and in addition the clusters detected in chromosome pair 5 [41]. Future cytogenetic analysis in *P. neildi* and *P. trinitatis* could be provides better compression about evolutionary dynamics of this chromosomal marker in this group.

The *P. distincta* karyotype showed NOR in chromosome pairs 1 and 9, corroborating a previous report [44], whereas *P. bahiana* showed NOR in pair 9, consistent with the findings of Barth et al. [42], as well as an additional marker in one homolog of pair 10 found in in two of eight specimens sampled in this study. Based in this scenario, Gruber et al. [44] proposed that the NOR in chromosome pairs 1 and 9 are conserved in the *P. burmesteri* group. The *P. bahiana* karyotype features reported in the present study provide novel insights into NOR evolution within this group. Based on the phylogenetic inferences [33], the *P. burmeisteri* group represents a monophyletic clade in which *P. bahiana* is a sister species of the remaining species within the group (*P. distincta*, *P. burmeisteri*, *P. iheringii* and *P. tetraploidea*). *Phyllomedusa sauvagii*, which remains unassigned to any phenetic group, is a sister species of the *P. burmeisteri* group. Here, we suggest that the NOR in chromosome pair 9 is the plesiomorphic karyotype condition. This idea is supported by the presence of this condition in *P. bahiana* and in the karyotype of *P. sauvagii*[39], both of which carry NOR on chromosome pair 9.

The NOR patterns of the species karyotyped in this work are consistent with the known notable characteristics of rDNA clusters in the *Phyllomedusa* genus; many cases of multiple NOR sites among the karyotyped species have been reported by other authors [39-44]. NOR position in the genome has been successfully used as a chromosomal marker in comparative cytogenetic studies in many vertebrates groups, and the possible role of NORs as hotspots of recombination during evolution has been widely discussed [3,9,48,49].

The origins of the ITSs detected in our analysis cannot be explained by assuming that ITSs represent remnants of structural chromosome rearrangements that occurred during the evolution of these karyotypes. Our arguments denote of expressive evolutionary chromosome conservation observed among the species of this genus with known karyotypes. If we consider the karyotype data in the light of the phylogeny proposed for the genus [33], the presence of ITSs in these karyotypes cannot be imputed to any traits of the chromosome rearrangements that are perceptible by classical cytogenetic. Indeed, the presence of ITSs in vertebrate genomes has been explained as a relic of the reorganization of chromosome architecture that occurred during the evolution of individual karyotypes. Through study of the comparative cytogenetic of many groups, it is possible to discern the remnants of chromosomal rearrangements from fission and/or fusion events [10,50,13-16] or chromosomal inversions [10,11] inferred from ITS signals. In amphibians, the presence of ITSs provides evidence of rearrangements that occurred during karyotype evolution in species of the Terrarana group [14] and has been invoked recently to explain the reduction in chromosome number in Dendropsophini [16].

Despite the fact that these suggestions are strongly supported by evidence from a number of organisms, the intrachromosomal telomeric repeats observed in the karyotypes of *P. vaillantii, P. distincta and P. bahiana* could be the result of amplifications of (TTAGGG)n repeats that occurred independently during the chromosomal evolution of these species. In opposition to the idea that the distribution of ITS in the karyotype represents remnants of ancient rearrangements, their distribution has usually been considered to be a result of the occurrence of double-strand breaks in the germ line [31]. Despite the fact that the precise molecular model accounting for these features is unclear, many studies have attributed the presence of widely distributed intrachromosomal ITSs to the insertion of telomeric DNA during the repair of double-strand breaks by the non-homologous end-joining pathway (NHEJ) [18,22,31,51]. Telomeric repeats are subject to evolutionary forces that can amplify the number of repeats or homogenize the repeat sequences according to the dynamics of evolution of the repetitive DNA sequences [19].

Non-terminal telomeric repeats were primarily detected in centromeric regions and coincided with regions of heterochromatin blocks (het-ITS). This interesting pattern has previously been reported in amphibians of the *Aplastodiscus* genus [26,28] and has been widely reported in rodents [24,52] and in plants of the *Solanum* genus [29,53].

ITS are frequently associated with heterochromatin regions, and previous investigations have shown that these repeats represent a primordial component of the repetitive DNA in cetacean [20,54], fishes [21] and rodents [22,27]. The conspicuous hybridized signals detected in some chromosome pairs such as the homologs of pair 13 of *P. vaillantii* indicate that (TTAGGG)n repeats represent a major motif in repetitive DNA.

## Conclusion

The presence of telocentric pairs in species of the *P. tarsius* group is an interesting feature observed within this genus: this species showed the same chromosomal number as another species, and the telocentric pairs found in species of this group are homologs of the subtelocentric pairs found in other karyotypes. In this case, it is possible that the short arms were lacking in the *P. tarsius* clade. Unlike the other species analyzed in this paper, the *P. tarsius* karyotype was the only karyotype that did not exhibit a hybridization signal for ITS. We suggest that additional experiments, including flow cytometric analysis and chromosomal painting should be conducted to better clarify the origins of this apparent autoapomorphy within the *P. tarsius* group.

In the karyotypes of *P. vaillantii, P. distincta and P. bahiana,* the most parsimonious explanation to the presence of ITS could be results of the amplifications of (TTAGGG)n repeats that occurred independently during the chromosomal evolution of these species. The results presented in this study will contribute to the understanding of the mechanisms of chromosomal evolution that have operated in *Phyllomedusa* genus, and provides evidences about the role of repetitive sequences in karyotypes diversification in vertebrates.

## Methods

We analyzed populations of *P. vaillantii*, *P. tarsius*, *P. distincta* and *P. bahiana* sampled from Brazil and Ecuador localities (Table 1). The collection of specimens from Brazil was authorized by SISBIO/ Instituto Chico Mendes de Conservação da Biodiversidade under number 20266–1. Specimens sampled in Brazil were deposited in the Museu de Zoologia “Prof. Adão José Cardoso” (ZUEC), at Universidade Estadual de Campinas, São Paulo, Brazil and the vouchers of populations sampled in Ecuador were deposited in the Museo de Zoología de la Pontificia Universidad Católica del Ecuador (QCAZ), Quito, Ecuador. The complete list of the species, localities sampled, number of individuals examined, and voucher numbers are provide in Table 1.

**Table 1:** **Species of *Phyllomedusa *** **analyzed, sample number (N) their respective sampling localities and voucher number**

**Species**	**N**	**Locality**	**Voucher**
*P. vaillantti*	01	Jacareacanga, Pará, Brazil.	ZUEC 15998
*P. vaillantti*	05	Yasuní, Provincia del Orellana, Ecuador	QCAZ 43241-43247
*P. vaillantti*	04	Porto Velho, Rondônia, Brazil	ZEUC 17035; 17036
*P. tarsius*	05	Reserva Ducke, Manaus, Brazil	ZUEC 16201-16204
*P. tarsius*	05	Yasuní/Provincia del Orellana, Ecuador	QCAZ 47276; 47278-47280
*P. distincta*	03	Iporanga, São Paulo, Brazil	ZUEC 17033-17035
*P. bahiana*	08	Alagoinhas, Bahia, Brazil	ZUEC 20656-20662

ZUEC: Museu de Zoologia “Prof. Dr. Adão Cardoso”, do Instituto de Biologia da Universidade Estadual de Campinas (UNICAMP), São Paulo, Brazil; QCAZ: Museo de Zoología de la Pontificia Universidad Católica del Ecuador (QCAZ), Quito, Ecuador.

The chromosomal preparations were obtained from intestinal and testicular cells of individuals previously treated with colchicine (2%) for 4 h following procedures modified from King and Rofe [55] and Schmid [56]. The mitotic metaphases were stained with 10% Giemsa to karyotyping determination. The identification of heterochromatic regions was performed using C-banding technical followed Sumner [57] with modifications. To better characterize the heterochromatic regions, C-banded chromosomes were stained with fluorochrome AT-specific DAPI and GC-specific Mytramycin (MM). We detected the NORs positions using the Ag-NOR method [58].

The physical map of the rDNA genes and telomeric sequences were detected by Fluorescent “in situ hybridization” (FISH) experiments using specific probes and protocols. To detected rDNA genes, we used 28S fragment isolated by Bruschi et al. [45]. The probe was PCR-labeled with digoxigenin, hybridized according to Viegas-Péquignot [59] and the hybridized signal was detected with an anti-digoxigenin antibody conjugated with rhodamine (Roche). Telomeric sequences was detected using the telomeric PNA probe (CCCTAA)_3 (_peptide nucleic acid - PNA -Applied Biosystems)_,_ kit performed following the manufactures’ manual. Metaphases were photographed under n Olympus BX-60 microscope and analyzed using the Image Pro-Plus software, version 4 (Media Cybernetics, Bethesda, MD, USA). The chromosomes were measured and the centromere index (CI), relative length (RL), and centromere ratio (CR) were estimated. The chromosomes were ranked and classified according to the scheme of Green and Sessions [60].

## Abbreviations

rDNA: Ribosomal DNA; ITS: Interstitial telomeric sequences; DAPI: 4 6-diamidino-2-phenylindole; NOR: Nucleolus organizer region; FISH: Fluorescence *in situ* hybridization.

## Competing interests

The authors declare that they have no competing interests.

## Authors’ contributions

DPB prepare and analysis of chromosomal data and drafted the manuscript. MR and ABZ helped prepare for the cytogenetic analysis. APL helped to collect and identify the specimens. SMRP designed and coordinated the study and helped draft the manuscript. All authors have read and approved the final manuscript.
